# Effects of Central Loop Length and Metal Ions on the Thermal Stability of G-Quadruplexes

**DOI:** 10.3390/molecules24101863

**Published:** 2019-05-15

**Authors:** Fengjin Hao, Yushu Ma, Yifu Guan

**Affiliations:** Department of Biochemistry and Molecular Biology, China Medical University, Shenyang 110122, China; haofengjin325@163.com (F.H.); haosiwendut@163.com (Y.M.)

**Keywords:** G-quadruplex, central loop length, thermal stability, entropy-driven, ionic effect

## Abstract

The central loop of G-quadruplex molecular beacons is a key element to sense target DNA or RNA sequences. In this study, circular dichroism spectroscopy (CD), thermal difference spectrum (TDS), non-denatured non-denaturing gel electrophoresis, and thermal stability analysis were used to investigate the effect of the central loop length on G-quadruplex features. Two series of G-quadruplexes, AG_3_TTAG_3_-(TTA)n-G_3_TTAG_3_T (*n* = 1–8) (named TTA series) and AG_3_TTTG_3_-(TTA)n-G_3_TTTG_3_T (*n* = 1–8) (named TTT series) were examined in K^+^ and Na^+^ solutions, respectively. CD and TDS spectral data indicated that TTA series adopted an antiparallel G-quadruplex structure in Na^+^ solution and a hybrid G-quadruplex structure in K^+^ solution respectively. TTT series exhibited a hybrid G-quadruplex structure in both Na^+^ and K^+^ solutions. UV melting curves indicated that the stability of G-quadruplex in both series was reduced by the elongation of central loop. Thermal stability analysis concluded that the G-quadruplex destabilization with long central loop is an entropy-driven process due to more flexible and longer central loops.

## 1. Introduction

**Figure 1:**
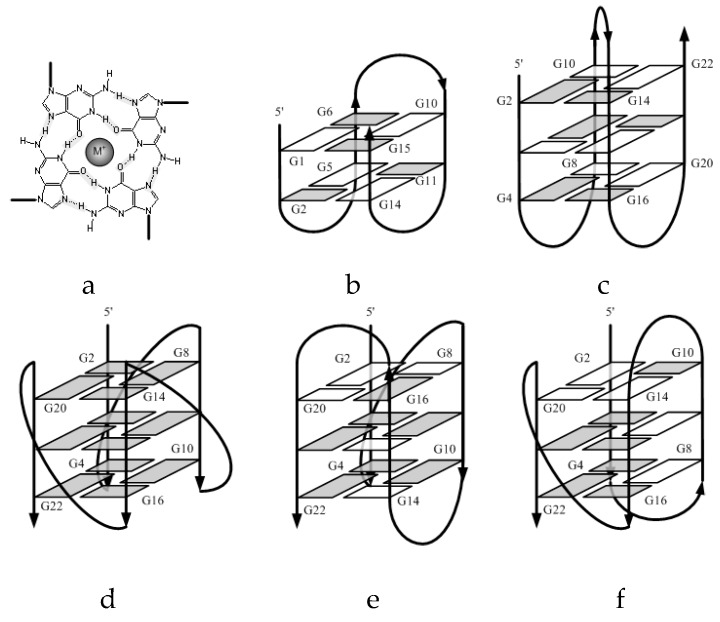
Schematic representations of intramolecular G-quadruplex (G4) structures: (**a**) a G-quartet plan with a coordinated ion, (**b**) antiparallel chair-type G4 of TBA sequence d[GGTTGGTGTGGTTGG] in K^+^ solution [39], (**c**) antiparallel basket-type G4 of telomeric sequence d[A(GGGTTA)_3_GGG] in Na^+^ solution, (**d**) parallel propeller-type G4 of telomeric sequence d[A(GGGTTA)_3_GGG] in a K^+^-containing crystal, (**e**) hybrid (3 + 1) type 1 G4 structure for d[TA(GGGTTA)_3_GGG] in K^+^ solution, and (**f**) hybrid (3 + 1) type 2 G4 structure for d[TA(GGGTTA)_3_GGGTT] in K^+^ solution [40]. The anti and syn guanine residues are colored grey and white, respectively.

Many guanine-rich sequences in human genome can fold into G-quadruplex (G4) structure. The G4 consists of stacked G-quartets (also named G-tetrads), each of which consists of four guanine residues connecting via Hoogsteen hydrogen bonds in a plane (Figure 1a). The G-quartets in G4 structure stack together linked with two side loops and one central loop. According to the strand polarities, the G4 structures are classified into a parallel, antiparallel or hybrid parallel/antiparallel folding topology (Figure 1b–f). These highly ordered G4 structures are found in telomeres [1,2,3], promoters [4,5], and 5′-UTR regions of mRNAs [6,7] of eukaryotic systems, suggesting their biological significance on gene expression [8,9,10,11,12]. Structure stability of G4 is usually influenced by the G-tract number [13,14,15], the loop of eukaryotic sequence [16,17,18,19,20,21,22], the loop size [10,13,14,16,17,18,19,20,21,22,23,24,25,26,27,28], and metal ions [29,30,31].

In addition to these native G4 structures, oligonucleotides with specially designed G4 sequences can be used for therapeutic [32,33,34] and bioanalytical [35,36,37] purposes. Several G4 sequences have been designed as molecular beacons to detect DNA and RNA sequences [38]. When the G4 molecular beacon adopts G4 structure, just as a folded stem-loop molecular beacon, no fluorescence signal can be measured. In contrast, when the central loop binds with the target DNA or RNA sequence, the unfolded G4 molecular beacon separates the fluorophore from the quencher, generating fluorescence signals remarkably. It has been revealed that the G4 molecular beacon can distinguish the perfect match and mismatch sequences unambiguously [38]. To optimize the targeting efficiency, several studies have examined the effects of the loops on the folding conformation and the stability of G4 structures, including the sequences of the loops [10,17,19], the lengths of each individual loop [19,27], and the total lengths of three loops [23]. In general, the G4 molecular beacons with a longer central loop (around ~20 nucleotides in length) can effectively hybridize their targets. However, the combined effects of longer central loops and the side loops on the stability of G4 structure are much less studied.

In the present work, two series of G-quadruplexes, AG3TTAG3-(TTA)n-G3TTAG3T (referred to as TTA series) and AG3TTTG3-(TTA)n-G3TTTG3T (referred to as TTT series) were used to explore the combined effects of the central loop and side loops on the G4 folding topology and its stability in K^+^ and Na^+^ buffers, respectively. The side loops of either TTT or TTA were chosen because the effect of one nucleotide difference between TTT and TTA had been noticed in previous experiments but not been investigated yet. In addition, K^+^ ions are believed to prefer the G4 formation, and Na^+^ ions can exert different effects. This study enriched our understanding about the G4 structure stability affected by different lengths of the central loop and side loops and provided us instructive guidelines to design the proper G4 molecular beacons.

## 2. Results

### 2.1. Formation of G-quadruplex

A number of G4 structures have been studied using X-ray crystallography and NMR spectroscopy in previous studies, and these structures have also been characterized by CD spectroscopic method. Consequently, empirical CD spectral signatures for different G4 folding topologies have been proposed. In general, an antiparallel G4 structure exhibits a negative peak at ~265 nm and two positive peaks: a minor one at ~240 nm and a major one at ~295 nm [41,42]. On the other hand, the parallel G4 structure is usually characterized by a negative peak at ~240 nm and a positive peak at ~265 nm in most cases [43,44]. When the G-rich sequences adopt a hybrid G4 structure, their CD spectra show a complex CD spectral profile: a negative peak at ~245 nm and a positive peak at ~295 nm with a broad shoulder at the lower wavelength side [44,45].

It has been advised that extreme care should be taken when classifying the G4 topology by using the empirical CD signatures since some inconsistence between CD data and the determined parallel G4 folding topology have been observed [19,46,47,48]. To ensure the assignment correctness, we re-collected CD spectra of several G4 oligonucleotides whose structures have been well defined with NMR method (Supplementary Materials Table S1). As shown, all antiparallel G4 structures exhibit one type of CD spectra whose features are consistent with the proposed empirical assignment (Figure S1c), and all hybrid G4 structures present another type of CD spectra (Figure S1b). However, parallel G4 structures display different CD results. CD spectra of c-myc [49,50], c-kit2 [51], RET20Tmer, RET21mer [52], and 93DEL [53] in K^+^ solution show a negative peak at ~240 nm and a positive peak at ~265 nm. On the other hand, T40214 demonstrates a CD signature of antiparallel G4 structure (Figure S1a) in K^+^ solution. Further examination indicates that these inconsistent results might be due to the ambiguous assignments of NMR structure of AS1411. One study reported an interlocked parallel G4 structurewhile another study exhibited an antiparallel G4 structure [54]. By excluding this one, overall, these CD spectral signatures have widely accepted as a convenient and quick method to distinguish different G4 structures.

**Figure 2:**
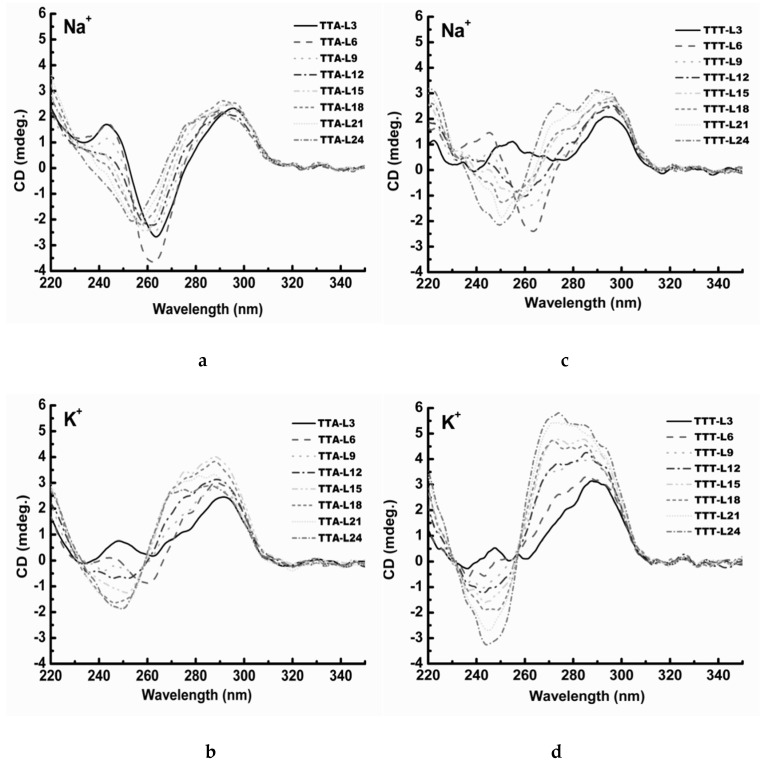
Circular dichroism (CD) spectra of TTA series and TTT series. (**a**) TTA series in 100 mM Na^+^ solution; (**b**) TTA series in 100 mM K^+^ solution; (**c**) TTT series in 100 mM Na^+^ solution; (**d**) TTT series in 100 mM K^+^ solution.

CD spectra of the TTA series in Na^+^solution are shown in Figure 2a. TTA-L3 illustrates a typical CD spectrum of an antiparallel G4 structure. As the central loop length becomes increased, the negative peak at 262 nm shows a stepwise leftward shift slightly, the positive peak at 295 nm remains unchanged, and the positive peak at 243 nm becomes diminished gradually. Overall, the shape of these CD spectra and the elliptic signals change very slightly. These results suggest clearly that the TTA series fold into an antiparallel G4 structure in Na^+^ solution, the central loop elongation only induces a minor local conformational change without changing the overall G4 topology. In K^+^ solution, TTA-L6 displays a CD spectrum of an antiparallel G4 structure (Figure 2b). However, TTA-L9 shows a typical CD spectrum of a hybrid G4 structure. As the central loop is elongated, the CD signature of hybrid G4 structure remains unchanged, the 247 nm peak becomes more negative, and the 275 nm shoulder and the 292 nm peak become more positive. These spectral changes suggest that the central loop elongation (longer than 9 nucleotides) led to the G4 molecules to display more chirality.

In Na^+^ solution, TTT-L6 shows a CD spectrum of an antiparallel G4 structure (Figure 2c). As the central loop increases, the CD spectral changes remarkably, indicating a transition from an antiparallel G4 structure to a hybrid G4 structure. The positive peak at 245 nm becomes more negative steadily, and the 295 nm peak becomes positive. Furthermore, an additional peak at 274 nm becomes apparent rapidly. In K^+^ solution, TTA-L6 shows a CD spectrum of a hybrid G4 structure (Figure 2d). As the central loop is elongated from TTT-L6 to TTT-L24, the 245 nm peak becomes more and more negative and the 290 nm peak becomes more and more positive. Again, the 274 nm shoulder grows rapidly into a new peak and even more intense than the 290 nm peak. The spectral changes could indicate that, in both K^+^ and Na^+^ solution, the central loop elongation makes the chirality of the TTT oligonucleotides more obvious and their more optical active generates stronger CD elliptic signals, while maintaining the overall hybrid G4 structure. TTA-L3 in K^+^ solution shows a similar CD spectrum to that of TTT-L3 in Na^+^ solution.

**Figure 3:**
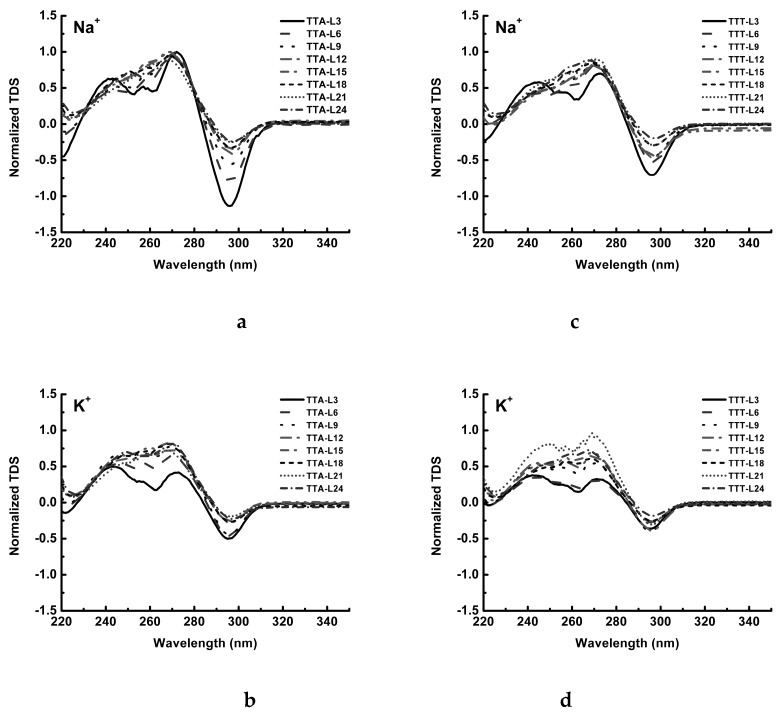
Thermal difference spectra of TTA series and TTT series. (**a**) TTA series in 100 mM Na^+^ solution; (**b**) TTA series in 100 mM K^+^ solution; (**c**) TTT series in 100 mM Na^+^ solution; (**d**) TTT series in 100 mM K^+^ solution.

Thermal difference spectrum (TDS) was also used to assess the formation of G4 structures. As indicated in Figure 3a, one huge negative peak at 295 nm and two positive peaks at 245 nm and 275 nm with different heights are the fingerprints of a G4 structure [39]. As the central loop of TTA series becomes longer, the magnitude of the negative peak at 295 nm is reduced significantly, and the valley between 245 nm and 275 nm becomes elevated (Figure 3a). In K^+^ solution, TTA-L3 also shows one negative peak at 295 nm and two positive peaks at 245 nm and 275 nm, and the rest TTA oligonucleotides having different central loop lengths show similar TDS profile (Figure 3b). In the case of the TTT oligonucleotides, they exhibit similar TDS profiles to that of TTA series in relevant Na^+^ and K^+^ solutions, respectively (Figure 3c,d).

### 2.2. Molecularity of G4 Structures

**Figure 4:**
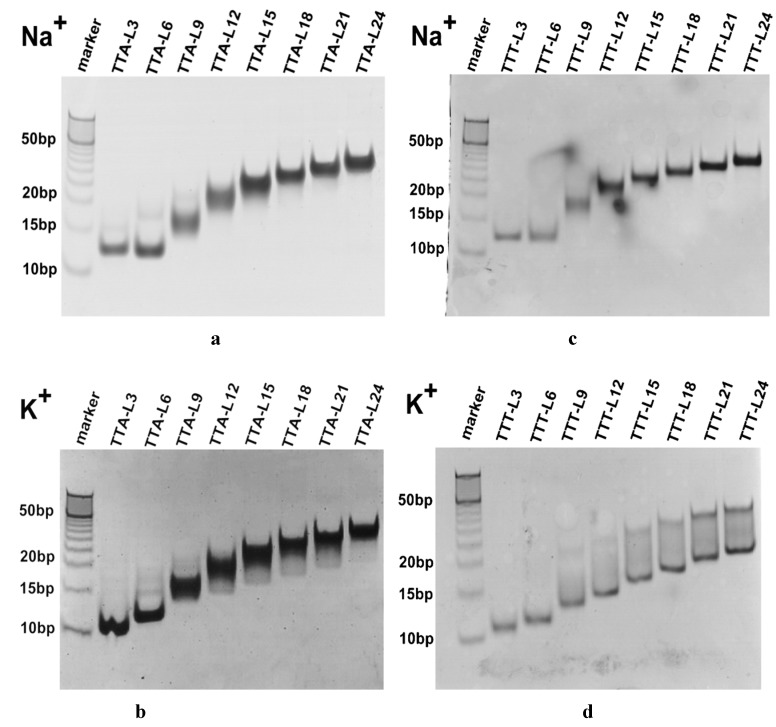
Non-denaturing electrophoresis analysis of TTA series and TTT series. (**a**) TTA series in 100 mM Na^+^-gel; (**b**) TTA series in 100 mM K^+^-gel; (**c**) TTT series in 100 mM Na^+^-gel; (**d**) TTT series in 100 mM K^+^-gel.

To determine the molecularity of G4 structures and to understand the structural variations induced by the central loop length in different ionic environments, we compared their mobility in non-denatured polyacrylamide gel. As shown in Figure 4a, in Na^+^-gel, TTA-L6 migrates at a mobility equivalent to the 12-bp marker, and as the central loop length increases, the TTA oligonucleotides demonstrate the retarded migrations. TTA-L24 moves at a rate approximately to the 37-bp marker. Interestingly, TTA-L3 moves slightly slower than TTA-L6. Most importantly, each oligonucleotide runs as a distinctive single band in Na^+^-gel, a suggestive of intramolecular conformation.

In K^+^-gel, all the TTA oligonucleotides run in a length-dependent manner: the longer the central loop, the slower the mobility (Figure 4b). The fastest (TTA-L3) band and the slowest (TTA-L24) band move at the rates equivalent to 10-bp and 37-bp markers, respectively. All the TTA species present one single band with the mobility compatible with its counterpart in the Na^+^-gel. Figure 4b shows an interesting phenomenon that when the central loop is longer than 9 nucleotides, the bands start to become smear. It might be correlated with the hybrid G4 structure.

The TTT sequences show a mobility pattern similar to that of the TTA species in Na^+^-gel (Figure 4a,c). Each TTT species migrates as a narrow single band at mobility proportional to the central loop length. It is observed again that TTT-L6 runs faster slightly than TTT-L3 (Figure 4c). In K^+^-gel, the TTT species demonstrate different mobility behavior. All bands migrate faster than their TTA counterparts in K^+^-gel (Figure 4b,d). Starting from TTT-L9, the band begins to become smear, and the slowest TTT-L24 shows the mobility similar to ~25-bp marker.

### 2.3. UV Melting Analysis of Structure Stability

**Figure 5:**
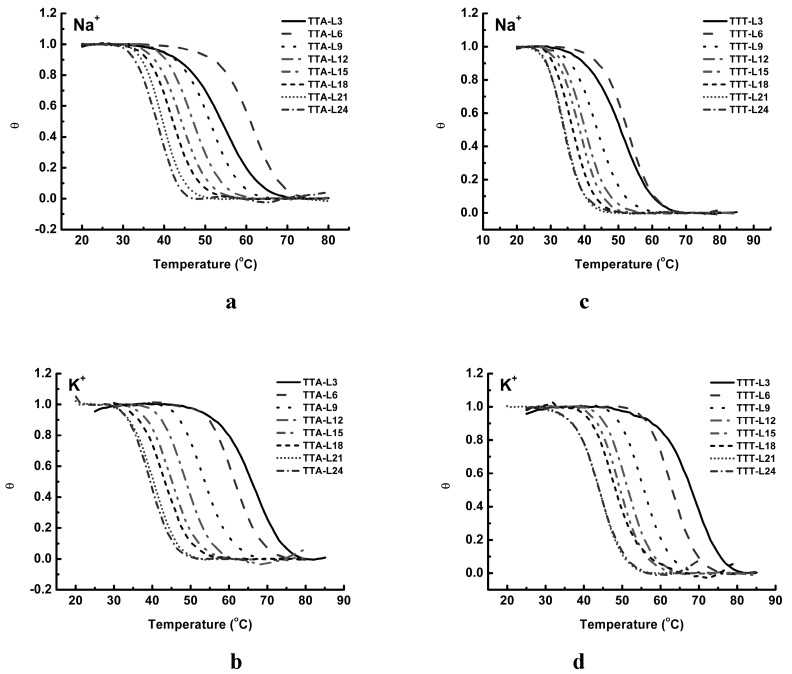
Folded fraction curves derived from UV melting curves of TTA series and TTT series. (**a**) TTA series in 100 mM Na^+^ solution; (**b**) TTA series in 100 mM K^+^ solution; (**c**) TTT series in 100 mM Na^+^ solution; (**d**) TTT series in 100 mM K^+^ solution.

The structure stabilities of these two series of oligonucleotides have been assessed using UV thermal analysis. The melting curves were recorded at 295 nm for all oligonucleotides. In addition, we also recorded their annealing curves at 295 nm [41]. To ensure that these unfolding and folding processes were accomplished under a condition of thermodynamic equilibrium, a slow heating/cooling rate at 0.3 °C/min was applied. To compare their thermal properties, their UV spectral curves were processed using the approach described. The folded fraction curves for each oligonucleotide in Na^+^ and K^+^ solution are shown in Figure 5, and their *T_m_*s and relevant thermodynamic parameters are summarized in Table 1 and Table 2.

In Na^+^ solution, the folded fraction curves of the TTA series suggest a two-phase thermal transition process [42]. The first derivative of each folded fraction curve also shows a single peak, confirming the two-phase transition (data not shown). Starting from TTA-L6, as the central loop length becomes longer, the folded fraction curves shift leftward gradually, and the melting temperatures decrease from 61.8 °C to 40.2 °C (Figure 4a). It is worth noticing that the folded fraction curve of TTA-L3 is slightly shallower than others, and its melting temperature is lower (53.8 °C) than that of TTA-L6 (61.8 °C). This feature of TTA-L3 might be correlated with the unusual phenomena in CD spectra of TTA-L3. At the heating/cooling rate of 0.3 °C/min, melting curves of shorter oligonucleotides are nearly reversible with their annealing curves, while oligonucleotides of a longer central loop show a small lagging between the melting curve and annealing curve (*T_m_* < 2–3 °C) (data not shown). In K^+^ solution, all the TTA sequences demonstrate similar folded fraction curves (Figure 5c). As the loop length increases, their melting curves shift leftward steadily, suggesting the effect of the longer central loop on the G4 stability.

**Figure 6:**
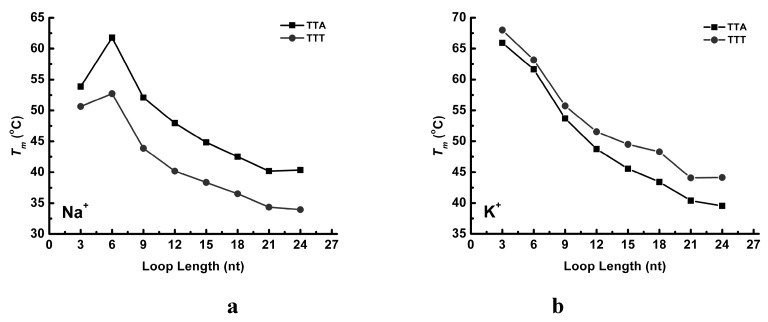
Correlation between the melting temperatures and the central loop lengths of TTA and TTT sequences in Na^+^ (**a**) and K^+^ (**b**) solutions, respectively.

Similarly, all the TTT sequences in Na^+^ solution undergo a two-phase thermal transition, and the central loop elongation shifts the folded fraction curves leftward (Figure 5c). Again, the melting temperature of TTT-L3 (50.6 °C) is slightly lower than that of TTT-L6 (52.7 °C). In K^+^ solution (Figure 5d), the TTT oligonucleotides show the folded fraction curves similar to that of the TTA series (Figure 5b). All these folded fraction curves do not show any signs of multiple-phase transition behavior. As a summary, the relationships between melting temperatures and loop length of TTA and TTT series are shown in Figure 6.

**Figure 7:**
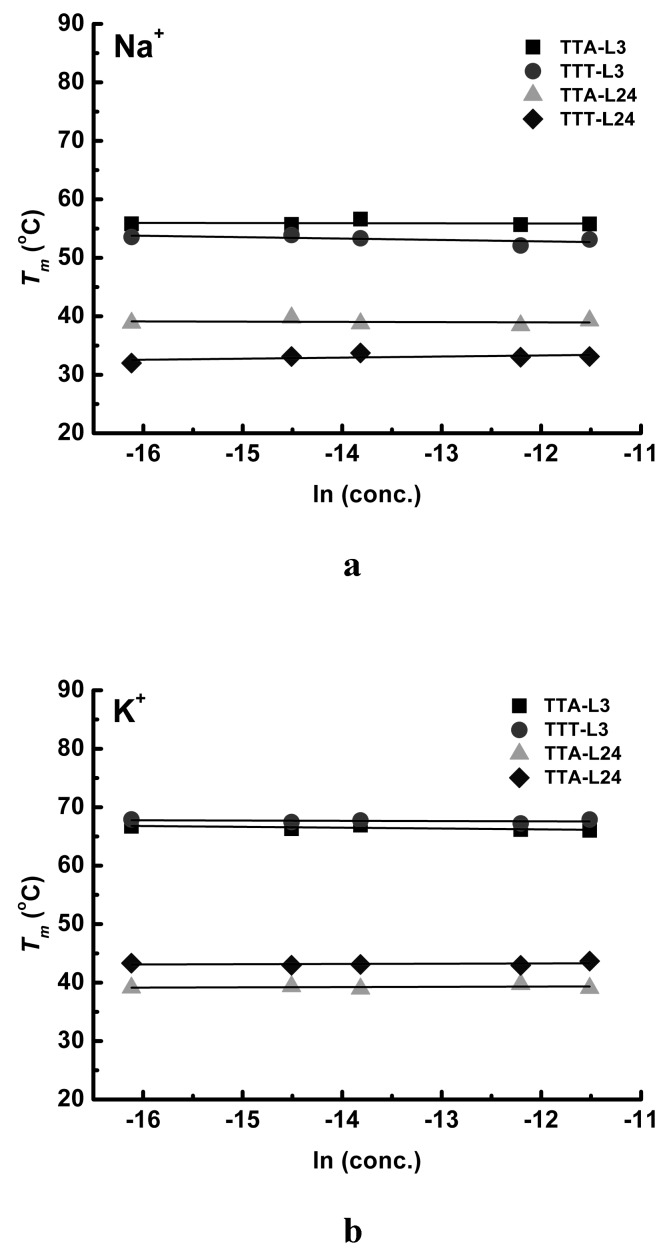
Concentration dependence of the Tm of TTA-L3 (triangle), TTA-L24 (circular), TTT-L3 (square) and TTT-L24 (diamond) in Na^+^ (**a**) and K^+^ (**b**) solutions, respectively. The concentrations of oligonucleotides are 0.1, 0.5, 1.0, 5.0, and 10.0 M.

To identify the molecularity of these G4 structures, the concentration-dependence of *T_m_* was examined. We investigate TTA-L3, TTT-L3, TTA-L24, and TTT-L24 at the concentrations of 0.5, 1, 2, 5, and 10 M, respectively. Figure 7 shows that the melting temperatures of the shortest (TTA-L3 and TTT-L3) and longest oligonucleotides (TTA-L24 and TTT-L24) do not change with respect to the oligonucleotide concentrations, indicating that the G4 structure of TTA and TTT series are an intramolecular species. The concentrations above 10.0 mM were not recorded because too intense absorption at 295 nm might raise the concern about the data accuracy.

Thermodynamics parameters (Δ*H*, Δ*S* and Δ*G*) of these G-quadruplexes have been derived from their UV melting curves using the reported procedure (Table 1). In the case of TTA series in Na^+^ solution, as the central loop is increased from (TTA)_2_ to (TTA)_8_, Δ*H* decreases monotonically whereas −*T*Δ*S* increases monotonically (Table 1). However, the decreased enthalpy is not large enough to compensate the increased entropy, resulting in a monotonically reduced Δ*G* and destabilizing the G4 structure. The increased entropy must be contributed from the increased flexibility of the elongated central loop of the G4 structure. TTT sequences in Na^+^ solution show a similar phenomenon: Δ*H* decreases monotonically and −*T*Δ*S* increases monotonically (Table 2). Again, the decreased Δ*G* results in the destabilization of the G4 structure.

In K^+^ solution, as the central loop of the TTA sequence is elongated, Δ*G* is reduced gradually, and both Δ*H* and *T*Δ*S* change moderately. However, the changes in Δ*H* and *T*Δ*S* do not follow any correlation with the loop length (Table 1). Similar results are also observed for TTT sequences in K^+^ solution (Table 2).

## 3. Discussion

### 3.1. Effect of the Central Loop on the G4 Structure

In present study, CD and TDS results have concluded that the TTA series in Na^+^ solution adopt the intramolecular antiparallel G4 structure, and the central loop elongation causes a minor change of elliptic signal consequently without changing the overall G4 topology. The same TTA series in K^+^ solution, however, present different CD spectra. TTA-L6 adopts an antiparallel G4 structure, while TTA-L9 shows a hybrid G4 structure, and further elongation of the central loop only enhances the chirality without changing the hybrid G4 structure (Figure 1b). Ambrus et al. have utilized NMR and CD to investigate the structure of telomeric sequence d[AAAG_3_TTAG_3_TTAG_3_TTAG_3_AA] (referred to as Tel26) in K^+^ and Na^+^ solutions, respectively [48]. NMR data have confirmed that this Tel26 sequence adopts a (3 + 1) hybrid G4 structure, and the CD spectra have shown a negative peak at 265 nm and a positive peak at 290 nm. In the current study, CD spectra of the TTA series show a high similarity to that of Tel26, suggesting a formation of hybrid G4 structure.

We have compared our results with a previous study of AG_3_TTAG_3_-T_n_-G_3_TTAG_3_T (*n* = 1~7) [19]. Their CD spectra indicated that the central loop containing one thymine residue folded into a parallel G4 structure in 100 mM K^+^ solution, whereas the central loop having more than three thymine residues adopted an antiparallel G4 structure. When the central loop had two thymine residues, the CD spectrum exhibited a signature of a hybrid G4 structure. To interpret these observations, this study has performed molecular dynamics simulation, and results showed that the central loops having either T or TT residues were too short to span the diagonal of the G-quartet in the intramolecular antiparallel G4 structure [19]. Further thermal analyses showed that the T_3_ central loop was the most stable G4 structure with *T_m_* of 68 °C, and when the loop length was increased to seven thymine residues, *T_m_* declined to 56 °C. All these results are in a good consistence with our study.

The TTT series demonstrate different G4 structures from the TTA series. The TTT-L6 shows a CD spectrum characterizing an antiparallel G4 structure in Na^+^ solution (Figure 2c). As the length of the central loop increases from (TTA)_2_ to (TTA)_8_, the positive peak at 250 nm evolves into a negative one, whereas the negative peak at 265 nm emerges to a visible positive peak at 270 nm. These results suggest that the TTT series undergo a structure conversion from an antiparallel G4 structure to a hybrid G4 structure (Figure 2c). In K^+^ solution, TTT-L6 shows a typical CD spectrum of the hybrid G4 structure, and as the central loop becomes longer, the CD peaks at 245 nm and 274 nm become intensified significantly (Figure 2d). These CD spectral changes imply that the central loop elongation might enhance the chirality of the G4 structure without altering the hybrid G4 folding topology.

As a further confirmation, we have obtained the thermal difference spectra (TDS) for all the TTA and TTT sequences (Figure 3). In comparison with that those representative TDS of G4 structures concluded by Mergny et al. [40], it is clear that all the TTA and TTT sequences maintain the G4 structure in Na^+^ and K^+^ solutions.

Another two similar sequences of G_3_TTTG_3_-T_n_-G_3_TTTG_3_ and G_3_TTAG_3_-T_n_-G_3_TTAG_3_ (*n* = 1–15) in K^+^ or Na^+^ solution have been compared previously [27]. Their CD spectra and TDS spectra showed that the G_3_TTTG_3_-TTT-G_3_TTTG_3_ sequence folded into an antiparallel G4 structure in Na^+^ solution while folding into a hybrid structure in K^+^ solution. Non-denaturing gel electrophoretic analysis showed that shorter central loops (*n* < 3) migrated more slowly than the longer loops (*n* > 4) due to the formation of the intermolecular parallel G4 structure. Also, when the loop was T or TT residues, their melting temperatures demonstrated a concentration-dependent behavior, confirming a formation of an intermolecular G4 structure. Similar results have also been observed for the TG_3_-T_n_-G_3_-T_n_-G_3_-T_n_-G_3_T sequence (*n* = 1 or 4) [20]. In the present study, non-denatured gel electrophoreses exhibit only one band in each lane (Figure 4a,c), implying that only one conformer is present for each TTA or TTT oligonucleotide in Na^+^ environment. However, in K^+^-gel, the TTT series become more smear. It could be an indicative that more conformers are co-present in K^+^-gel.

As mentioned, Bourdoncle et al. have investigated the utility of the G4 molecular beacon of G_3_TTAG_3_-loop-G_3_TTAG_3_, where the loop was 3, 6, 15, 18, and 21 nucleotides long, respectively [38]. In K^+^ or Na^+^ solution, these oligonucleotides folded into an antiparallel G4 structure determined by CD, and the increased central loop length destabilized their G4 structure. They prepared a G4 molecular beacon having a central loop of 13 nucleotides long, and demonstrated its capability of distinguishing the complementary target sequence from those with one mismatch. However, it is still a puzzle that why only two nucleotide substitutions (each in loop1 and loop3) could cause such significant differences. It could be attributed to the intramolecular interactions since adenine base has a fused structure of five-membered ring and six-membered ring, whereas thymine base has only a six-membered ring structure. When they reside in loop1 and loop3, their interactions with surroundings could be different for unknown reasons.

### 3.2. Effect of Cations

It has been known that cations play an important role in determining the G4 folding topology and structure stability. Oligonucleotide AG_3_TTAG_3_-TTA-G_3_TTAG_3_ in 100 mM Na^+^ solution adopted a basket-type antiparallel G4 structure [43], while in K^+^ solution, it existed as a hybrid G4 structure [43,44,48]. However, its crystal structure in the presence of K^+^ ions was determined to be a propeller-type parallel G4 structure [45]. It was also found that two similar sequences TAG_3_TTAG_3_-TTA-G_3_TTAG_3_ and TAG_3_TTAG_3_-TTA-G_3_TTAG_3_TT adopted two different hybrid G4 structures in K^+^ solution [46]. Similar phenomena have also been observed for G_3_TTAG_3_-TTA-G_3_TTAG_3_ and G_3_TTTG_3_-TTT-G_3_TTTG_3_ in Na^+^ and K^+^ solutions, respectively [14]. Thorough analyses have concluded that only the cations with the radius of 1.3 Å–1.5 Å could stabilize the antiparallel chair-type G4 structure by being sandwiched by two G-quartets, while other ions were either too small or too large to fit the space and to stabilize the G4 structure [31].

In the current study, the TTA series in Na^+^ solution exhibits an antiparallel G4 structure regardless the length of the central loop, while in K^+^ solution, they exist as a hybrid G4 structure. On the other hand, the TTT series of longer central loop adopt a hybrid G4 structure in Na^+^ and K^+^ solutions. These results, in general, are in a good agreement with the data in previous studies.

The effect of cations on the G4 structure stability has also been assessed using UV absorption analysis by several groups. When dissolved in cation buffer, the sequence G_3_-TTA-G_3_-loop-G_3_-TTA-G_3_ (loop = TCCTTTGTTTGT) showed the structure stability trend characterized by *T_m_*: LiCl (15 °C) < NaCl (46 °C) < KCl (55 °C) [38]. In another previous study, when the central loop of G_3_-TTA-G_3_-T_n_-G_3_-TTA-G_3_ was 3, 6, 9, and 12 nucleotides long, their melting temperatures were 65.3, 62.3, 54.0, and 48.0 °C in K^+^ solution, and 56.6, 60.8, 52.8, and 46.8 °C in Na^+^ solution, respectively [27]. Many studies have reached a similar conclusion that K^+^ ions have more profound effect than other ions on the G4 structure stabilization, and longer loops have lower thermal stability. This empirical rule was also suitable for many other G4 forming sequences [10,14,17,27] and even for variable sequences of the central loop [22]. Our results exhibit an interesting feature. The TTT series have higher *T_m_*s in K^+^ solution than that in Na^+^ solution by ~10 °C. However, the TTA series show that the *T_m_*s in K^+^ solution are higher than that in Na^+^ solution only by a small margin (Table 1).

It is interesting to notice that although TTA series and TTT series demonstrate many differences in folding topology, thermal stability, and thermodynamic parameters in K^+^ and Na^+^ solutions, they are different from each other by only two nucleotides (one in loop1 and one in loop3). Previous study has observed that when the loop residues were replaced by non-nucleoside linkers, G_3_(TTAG_3_)_3_ sequence in K^+^ or Na^+^ solution exhibited characteristic CD spectra of parallel G4 structure [10], demonstrating the ionic effects on the G4 structure. A molecular dynamics simulation study of AG_3_TTAG_3_-loop-G_3_TTAG_3_T (loop length = 1–7 thymine residues) has shown that interactions within loops could stabilize the preferential antiparallel G4 structure with loop lengths of three or more, while such stabilizing interaction are less present in parallel G4 structure. In the case of antiparallel G4 structure, two TTA lateral loops could add additional stacking interactions of the diagonal loop residues [19]. In comparing these reported results, it is speculated that interactions within the loops as well as the interactions between the loop and surrounding ions might have certain influencing effects in our case. In particular, Figure 4d shows smear bands when the central length becomes longer, which could be due to the co-presence of two hybrid G4 structures of TTT series in K^+^ environment. Unfortunately, CD data cannot distinguish hybrid type 1 G4 structure and hybrid type 2 G4 unambiguously. More studies are needed to uncover the true mechanism in future.

### 3.3. Thermal Properties of G4 Structures

Examination of the folded fraction curves in Figure 4 shows that as the central loop becomes elongated, the G4 structure stabilities decreased. This trend is consistent with previous reports [27]. When the central loop of G_3_-TTA-G_3_-T_n_-G_3_-TTA-G_3_ was 3, 6, 9, and 12 nucleotides long, their melting temperatures were 65.3, 62.3, 54.0, and 48.0 °C the in K^+^ solution, and 56.6, 60.8, 52.8, and 46.8 °C in Na^+^ solution, respectively [27]. Investigation of a similar sequence of G_3_-TTT-G_3_-T_n_-G_3_-TTT-G_3_ in Na^+^ and K^+^ solutions demonstrated similar results. Our data show a good consistence with these experimental results (Table 1).

**Figure 8:**
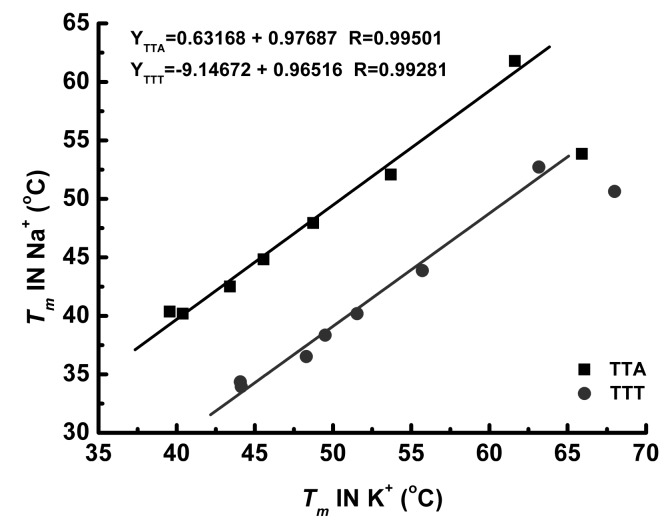
Correlation between melting temperatures of TTA and TTT sequences in 100 mM Na^+^ and in 100 mM K^+^ solutions, respectively.

When plotting the melting temperatures with respect to the central loop length, Figure 6 show a monotonic linear decline in K^+^ solution for both the TTA and TTT sequences. However, the curves of TTA and TTT sequences in Na^+^ solution shows that TTT-L6 and TTA-L6 have the highest *T_m_*. This result is in a good consistence with a previous study of G_3_TTTG_3_-loop-G_3_TTTG_3_ and G_3_TTAG_3_-loop-G_3_TTAG_3_ [27]. The loop length varied from 1 to 15 nucleotides long, and the correlation curves showed that the *T_m_* peak appeared when *n* = 4~5 for both sequences [27]. We also evaluated the length-dependent effect by graphing *T_m_*s in Na^+^ solution versus *T_m_*s in K^+^ solution. As illustrated in Figure 8, these *T_m_*s in Na^+^ solution are highly correlated with *T_m_*s in K^+^ solution (R = 0.997 for TTA sequences and R = 0.993 for TTT sequences) when excluding the L3 sequences. Table 1 shows the calculated van’t Hoff data (enthalpy and entropy) for each sequence. In Na^+^ solution, Δ*H* decreases monotonically and −*T*Δ*S* increases monotonically as the central loop of the TTA series is increased. Further examination of these data showed that the decreased enthalpy is not great enough to compensate the increased entropy, resulting in the G4 structure destabilization characterized by the monotonically reduced Δ*G*. The increased entropy must be contributed from the increased flexibility of the elongated central loop of the G4 molecular beacon. The TTT sequences in Na^+^ solution demonstrated a similar trend.

These entropic changes should be closely correlated with the G4 folding topologies. When folding into an intramolecular parallel G4 structure, all three loops (L1 loop, central loop, and L3 loop) serve as propeller linkers to connect the top G-quartet and the bottom G-quartet (Figure 1d). On the other hand, to form an intramolecular antiparallel G4 structure, all three loops only need to connect two guanine residues in the same G-quartet, either the top or the bottom as lateral linkers (Figure 1b,c). Thus, these three loops could be much less flexible for the intramolecular parallel G4 structure than the intramolecular antiparallel one due to the loop constrains. This flexibility effect becomes more profound for longer central loops, and makes different contributions to the enthalpy and entropy (Table 1 and Table 2).

In K^+^ solution, as the central loop of the TTA sequence is elongated, Δ*G* is reduced gradually due to the changes of both Δ*H* and *T*Δ*S*. However, the changes in Δ*H* and *T*Δ*S* do not show any trend with the loop length (Table 1). Similar results are also observed for TTT sequences in K^+^ solution (Table 2). To understand the unexpected data, we have compared with Bourdoncle’s study. As mentioned above, they used the G_3_TTAG_3_-loop-G_3_TTAG_3_ sequence as a model of G4 molecular beacon. The central loops with 3, 6, 15, 18, and 21 nucleotides long have been studied in KCl and NaCl solutions, respectively [38]. As the loop length increased, their thermal stability (Δ*G* and *T_m_*) was reduced steadily. However, both Δ*H* and *T*Δ*S* did not show any direct relationship with the central loop lengths.

There have been debates about the contributions of enthalpy and entropy to the stability of G4 structure. However, no conclusive remarks have been derived. Previous study of molecular dynamic simulation proposed that the enthalpic contribution favors the antiparallel G4 structure, whereas the entropic contribution favors the parallel G4 structure [19]. There is no such empirical rule for the hybrid G4 structure. Zhang et al. have observed that regardless the length of each individual loop, any G4 sequences with a total loop length of less than five nucleotides showed typical CD spectra of parallel G4 topology, and as the total length increases, there is a trend of transition from the parallel to the antiparallel G4 structure [24]. Thermodynamic calculations have derived an empirical rule: addition of one nucleotide into the loop led to a significant decrease in both *T_m_* and Δ*G* at 37 °C when the total loop length is less than five nucleotides, while any increase in loop lengths does not significantly affect the stability when the total loop length is more than five nucleotides [23]. In the study of G_3_TTTG_3_-loop-G_3_TTTG_3_ and G_3_TTAG_3_-loop-G_3_TTAG_3_ (loop length = 1–12 thymine residues), a close correlation between the total loop length and *T_m_* was established: adding one nucleotide resulted in a 2 °C drop or ~0.3kcal/mol in Δ*G* in K^+^ solution. However, this trend was less clear in Na^+^ solution [27]. We have made a further comparison with a study of AG_3_TTAG_3_-loop-G_3_TTAG_3_T (loop length = 1–7 thymine residues) in K^+^ solution. As the central loop length increased from one thymine to seven thymine residues, CD spectra exhibited a clear transition from the parallel to the antiparallel G4 structure [19]. Molecular dynamic simulations have been performed to assess the central loop structures using stacking and hydrogen-bonding abilities. The calculated thermodynamic parameters showed that the antiparallel G4 structures were generally favored.

## 4. Materials and Methods

### 4.1. Preparation of Oligonucleotides

All the oligonucleotides were synthesized in Sangon Biotech (Shanghai) Co., Ltd. (Shanghai, China). The stock solutions of AG_3_TTAG_3_-(TTA)_n_-G_3_TTAG_3_T (*n* = 1–8) (named TTA series) and AG_3_TTTG_3_-(TTA)_n_-G_3_TTTG_3_T (n = 1–8) (named TTT series) (Table 3) were prepared with ddH_2_O at the concentration of 100μM and stored at −20 °C. All samples were denatured at 95 °C for 5 min and then cooled down slowly to the room temperature.

### 4.2. Circular Dichroism Spectroscopy

CD spectra were recorded on a spectropolarimeter (JASCO J-810, Tokyo, Japan) over a wavelength range of 220 nm–350 nm at 25 °C, with the instrument scanning speed of 500 nm/min, a response time of 0.5 s, a data pitch of 0.5 nm, and a band width of 2.0 nm. The sample in a quartz cell of 0.1 cm path length was placed in a thermo-stable cell holder. The presented spectra were an average of three independent scans with baseline-correction via subtracting the signal contributions of the buffer. The 5 μM oligonucleotide samples were prepared for CD measurement in Na^+^ buffer and K^+^ buffer respectively. The Na^+^ buffer contains 10 mM NaH_2_PO_4_ and 90 mM NaCl and the K^+^ buffer contains 10 mM KH_2_PO_4_ and 90 mM KCl. A slow melting-annealing cycle was performed prior to each CD experiment.

### 4.3. UV Melting Measurement

UV melting curves of the oligonucleotides were recorded at 295 nm on a Cary 100 UV-Vis spectrophotometer equipped with a temperature control accessory (Varian, Melbourne, Australia). Spectra were recorded between 20 °C and 90 °C with 1 °C increment during heating and cooling processes. A slow heating/cooling rate of 0.3 °C/min was applied to ensure the thermal transition under an equilibrium condition. The denaturation and renaturation curves for data analysis were an average of three independent measurements. The samples were prepared in the buffer of 10 mM KH_2_PO_4_ and 90 mM KCl (pH 7.4) or the buffer of 10 mM NaH_2_PO_4_ and 90 mM NaCl (pH 7.4) respectively. Samples in capped quartz cuvettes with 1.0 cm path length were used in the measurement. The hysteretic behavior of the melting/annealing process was used to evaluate the G4 structure formation. In order to evaluate the molecularity of the G4 structure, the UV absorbance spectra of TTA-L3 and TTA-L24 as well as TTT-L3 and TTT-L24 at the concentration of 0.1, 0.5, 1.0, 5.0, and 10.0 μM in either Na^+^ or K^+^ buffer were recorded.

### 4.4. Thermodynamic Parameters Calculation

The UV absorbance spectra recorded at 295 nm were analyzed using software installed in Cary 100 UV-Vis spectrophotometer (Shimadzu, Japan). This software is designed based on the principle of nonlinear least square curve fitting and Van’t Hoff plot method [40]. First, the best-fit baselines of melting curves before and after thermal transition were obtained using the nonlinear least-squarer fitting approach, and then normalized using the formula: *θ*_T_ = [A(*T*) − A_U_(*T*)]/[A_F_(*T*) − A_U_(*T*)], where *θ* is the folded fraction of G4 at a given temperature, A(*T*), A_U_(*T*), and A_F_(*T*) represent the UV absorbance at a given temperature, in the unfolded format and in the folded format at 295nm. The melting/annealing data were normalized using the formula: F_F_(θ) = (Y − Ymin)/(Ymax − Ymin), where was the fraction of the folded G4 at a given temperature, Y was the signal at 295 nm. The normalized profiles (fraction folded profiles) were then used to determine the *T*_m_ (melting temperature) or *T*_a_ (annealing temperature) values at *θ* = 0.5 [41]. These normalized melting curves were used to calculate the thermodynamic parameters (Δ*H*, Δ*S,* and Δ*G*) when the concentration of oligonucleotide, the molecularity and the temperature were given [42]. In general, the analysis assumes that Δ*H* is independent of temperature, which means that the heat capacity change (Δ*Cp*) during the thermal transition is zero. Standard Δ*H* and Δ*S* was used to determine the Gibbs energy and the association constant (*K_a_*). *K_a_* was obtained using the formula *K_a_* = *θ*/(*1* − *θ* ), and ln(*K_a_*) was plotted as a function of 1/*T* in K^−1^. By using the equation of free Gibbs energy Δ*G* = −*RT*ln(*K_a_*) = Δ*H* − (*T*Δ*S*), ln(*K_a_*) could be equal to −(Δ*H*/*R*)(1/T) + Δ*S*/*R*. The plot of ln(*K_a_*) versus 1/*T* is a straight line which a slope of −Δ*H*/*R* and an intercept on Y axis of Δ*S*/*R*. In the current study, all the original experimental curves for data processing are an average of at least three independent measurements and the accuracy of *T_m_* and *T_a_* values was within ±0.1 °C.

### 4.5. Thermal Difference Spectrum (TDS)

Absorption spectra were recorded on Cary 100 UV-Vis spectrophotometer, in the range from 200 nm to 350 nm, with a scan speed of 600 nm/min and a spectral increment of 1 nm. The spectra were collected at 90 °C and 15 °C, which corresponded to the unfolded and folded states respectively. Thermal differential spectra were obtained via subtracting the absorption spectra at 15 °C from that at 90 °C. The difference spectra were normalized by dividing the data by its maximum y-value.

### 4.6. Non-Denatured Gel Electrophoresis

Oligonucleotides of 5 μM were incubated in the buffer (10 mM Tris-HCl, pH 7.4) with 100 mM KCl or 100mM NaCl at room temperature for 1 h. The electrophoresis experiments were performed at 4 °C in a 20% non-denatured polyacrylamide gel, which contained 100 mM KCl or NaCl. Gels were stained with Stains-all (Sigma-Aldrich, USA) in the dark and destained in water, and the images were recorded using a Personal Scanner (Model Z320, Fangzheng, China).

## 5. Conclusions

In this study, we used CD spectroscopy, thermal difference spectrum, non-denatured gel electrophoresis and thermodynamic analysis to investigate how the central loop length influences the folding topology and the structure stability of G-quadruplexes in K^+^ and Na^+^ ionic environments. Experimental results demonstrated that the TTA series adopted an antiparallel G4 structure in Na^+^ solution and a hybrid G4 structure in K^+^ solution, respectively. On the other hand, the TTT series exhibited a hybrid G4 structure in both Na^+^ and K^+^ solutions. In both series, the central loop elongation reduced the G4 structure stability significantly. Thermodynamic data indicated that the G4 structure destabilization caused by central loop elongation of TTT and TTA series in Na^+^ solution is an entropy-driven process. These results and conclusions enrich our understanding about the G-quadruplex formation and highlight the application potential of these G4 structures in biosensing and gene expression regulation. This inconsistence highlights the necessity for more thorough investigations regarding the CD signature assignments for different G4 folding topology.

## Figures and Tables

**Table 1:** Thermal dynamic parameters (Δ*H*, *T*Δ*S* and Δ*G* (25 °C)) of TTA sequences in 100 mM NaCl or 100 mM KCl solutions, respectively.

Species	Na^+^				K^+^			
	Δ*H* (kJ/mol)	−*T*Δ*S* (kJ/mol)	Δ*G* (kJ/mol)	*T_m_* (°C)	Δ*H* (kJ/mol)	−*T*Δ*S* (kJ/mol)	Δ*G* (kJ/mol)	*T_m_* (°C)
TTA-L3	−198.0 (±0.7)	180.7 (±0.6)	−17.3 (±0.1)	53.8 (±0.1)	−221.3 (±6.6)	194.7 (±5.8)	−26.6 (±0.7)	65.9 (±0.0)
TTA-L6	−212.2 (±5.4)	188.9 (±4.9)	−23.4 (±0.5)	61.8 (±0.0)	−300.3 (±4.5)	267.4 (±3.9)	−32.9 (±0.6)	61.6 (±0.2)
TTA-L9	−243.9 (±2.9)	223.8 (±2.7)	−20.2 (±0.2)	52.1 (±0.0)	−265.4 (±2.9)	242.0 (±2.7)	−23.3 (±0.2)	53.7 (±0.0)
TTA-L12	−261.8 (±1.1)	243.2 (±1.1)	−18.7 (±0.1)	47.9 (±0.0)	−299.6 (±3.7)	277.4 (±3.4)	−22.2 (±0.3)	48.7 (±0.0)
TTA-L15	−283.9 (±0.8)	266.3 (±0.8)	−17.7 (±0.0)	44.8 (±0.0)	−285.2 (±2.8)	266.8 (±2.6)	−18.4 (±0.2)	45.6 (±0.1)
TTA-L18	−294.1 (±2.3)	277.8 (±2.1)	−16.3 (±0.2)	42.5 (±0.0)	−275.8 (±1.7)	259.6 (±1.7)	−16.2 (±0.1)	43.4 (±0.1)
TTA-L21	−304.5 (±2.1)	289.7 (±1.9)	−14.8 (±0.2)	40.2 (±0.0)	−301.2 (±3.7)	286.4 (±3.5)	−14.8 (±0.2)	40.4 (±0.0)
TTA-L24	−329.2 (±0.7)	313.0 (±0.8)	−16.1 (±0.0)	40.4 (±0.0)	−310.0 (±6.7)	295.4 (±6.3)	−14.5 (±0.3)	39.6 (±0.0)

**Table 2:** Thermal dynamic parameters (Δ*H*, *T*Δ*S,* and Δ*G* (25 °C)) of TTT sequences in 100 mM NaCl or 100 mM KCl solutions, respectively.

Notation	Na^+^				K^+^			
	Δ*H* (kJ/mol)	−*T*Δ*S* (kJ/mol)	Δ*G* (kJ/mol)	*T_m_* (°C)	Δ*H* (kJ/mol)	−*T*Δ*S* (kJ/mol)	Δ*G* (kJ/mol)	*T_m_* (°C)
TTT-L3	−194.4 (±1.3)	179.2 (±1.2)	−15.3 (±0.1)	50.6 (±0.0)	−217.9 (±2.7)	190.5 (±2.4)	−27.4 (±0.3)	67.9 (±0.1)
TTT-L6	−244.8 (±3.4)	224.1 (±3.1)	−20.7 (±0.3)	52.7 (±0.0)	−309.7 (±2.1)	274.5 (±1.9)	−35.2 (±0.2)	63.2 (±0.0)
TTT-L9	−235.2 (±2.0)	221.1 (±1.8)	−14.0 (±0.2)	43.9 (±0.0)	−295.8 (±2.5)	268.0 (±2.3)	−27.7 (±0.3)	55.7 (±0.0)
TTT-L12	−251.2 (±0.8)	238.9 (±0.8)	−12.2 (±0.1)	40.2 (±0.0)	−290.9 (±1.6)	267.1 (±1.5)	−23.8 (±0.1)	51.5 (±0.0)
TTT-L15	−268.5 (±1.9)	256.9 (±1.7)	−11.5 (±0.2)	38.4 (±0.1)	−290.5 (±1.1)	268.4 (±1.1)	−22.1 (±0.1)	49.5 (±0.0)
TTT-L18	−287.2 (±4.3)	276.4 (±4.2)	−10.7 (±0.2)	36.5 (±0.1)	−229.0 (±2.3)	212.5 (±2.2)	−16.5 (±0.2)	48.3 (±0.0)
TTT-L21	−303.8 (±2.9)	294.4 (±2.8)	−9.3 (±0.2)	34.4 (±0.1)	−276.9 (±1.1)	260.5 (±1.0)	−16.4 (±0.0)	44.1 (±0.0)
TTT-L24	−252.1 (±1.8)	245.1 (±1.7)	−7.0 (±0.1)	33.9 (±0.0)	−265.2 (±3.0)	249.2 (±2.8)	−15.9 (±0.1)	44.1 (±0.1)

**Table 3:** Two series of oligonucleotides used in this study.

Species	Sequence	Central Loop Length
TTA-L3	AG_3_-TTA-G_3_-(TTA)_1_-G_3_-TTA-G_3_T	3
TTA-L6	AG_3_-TTA-G_3_-(TTA)_2_-G_3_-TTA-G_3_T	6
TTA-L9	AG_3_-TTA-G_3_-(TTA)_3_-G_3_-TTA-G_3_T	9
TTA-L12	AG_3_-TTA-G_3_-(TTA)_4_-G_3_-TTA-G_3_T	12
TTA-L15	AG_3_-TTA-G_3_-(TTA)_5_-G_3_-TTA-G_3_T	15
TTA-L18	AG_3_-TTA-G_3_-(TTA)_6_-G_3_-TTA-G_3_T	18
TTA-L21	AG_3_-TTA-G_3_-(TTA)_7_-G_3_-TTA-G_3_T	21
TTA-L24	AG_3_-TTA-G_3_-(TTA)_8_-G_3_-TTA-G_3_T	24
TTT-L3	AG_3_-TTT-G_3_-(TTA)_1_-G_3_-TTT-G_3_T	3
TTT-L6	AG_3_-TTT-G_3_-(TTA)_2_-G_3_-TTT-G_3_T	6
TTT-L9	AG_3_-TTT-G_3_-(TTA)_3_-G_3_-TTT-G_3_T	9
TTT-L12	AG_3_-TTT-G_3_-(TTA)_4_-G_3_-TTT-G_3_T	12
TTT-L15	AG_3_-TTT-G_3_-(TTA)_5_-G_3_-TTT-G_3_T	15
TTT-L18	AG_3_-TTT-G_3_-(TTA)_6_-G_3_-TTT-G_3_T	18
TTT-L21	AG_3_-TTT-G_3_-(TTA)_7_-G_3_-TTT-G_3_T	21
TTT-L24	AG_3_-TTT-G_3_-(TTA)_8_-G_3_-TTT-G_3_T	24

## Supplementary Materials

The following are available online, Figure S1: CD spectra of 16 G-4 forming sequences. The oligonucleotide samples were prepared at the final concentration of 5 μM in a buffer containing 10 mM phosphate buffer at pH 7.4. For clarity, the 16 GROs have been presented in parallel G-quadruplex (a), hybrid G-quadruplex (b), basket-type antiparallel G-quadruplex (c), and chair-type antiparallel G-quadruplex (d), respectively. (e) Examples of the exception of chair-type antiparallel G-quadruplexes; Table S1: Nucleotide sequences used for the CD spectral study; Table S2: The sequences studied in this manuscript.
