# Supplementary material for: Effects of Central Loop Length and Metal Ions on the Thermal Stability of G-Quadruplexes
Source: Molecules. 2019 May 15;24(10):1863. doi: 10.3390/molecules24101863 (PMC6571788; doi:10.3390/molecules24101863)
Supplement: Supplementary file 1 [file molecules-24-01863-s001.pdf]

Supplementary Materials

Figure S1.

CD spectra of 16 G-4 forming sequences. The oligonucleotide samples were prepared at the final concentration of 5 μM in a buffer containing 10mM phosphate buffer at pH 7.4. For clarity, the 16 GROs have been presented in parallel G-quadruplex (a), hybrid G-quadruplex (b), basket-type antiparallel G-quadruplex (c), and chair-type antiparallel G-quadruplex (d), respectively. (e) Examples of the exception of chair-type antiparallel G-quadruplexes.

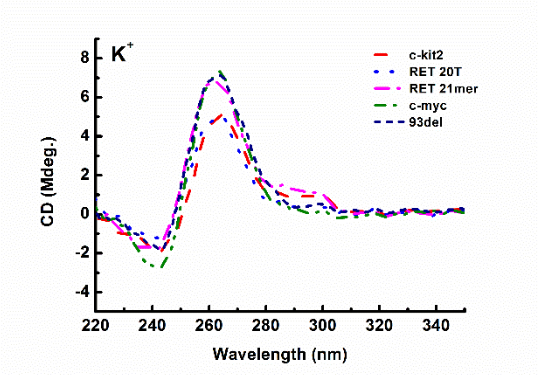

a

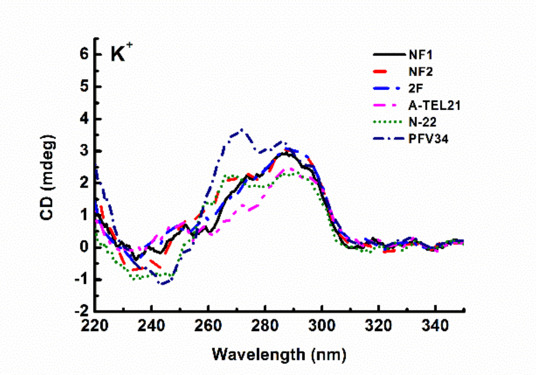

b

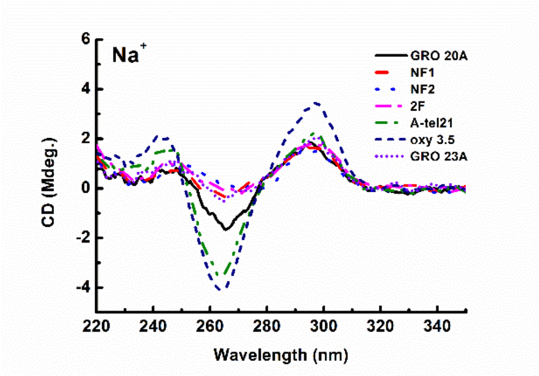
Na+
GRO 20A
NF1
NF2
2F
A-tel21
oxy 3.5
GRO 23A
CD (Mdeg.)
Wavelength (nm)

c

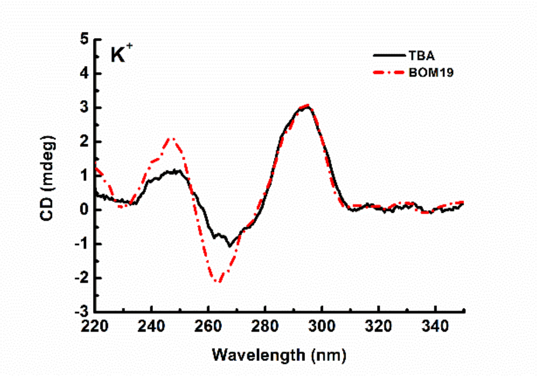
K+
TBA
BOM19
CD (mdeg)
Wavelength (nm)

d

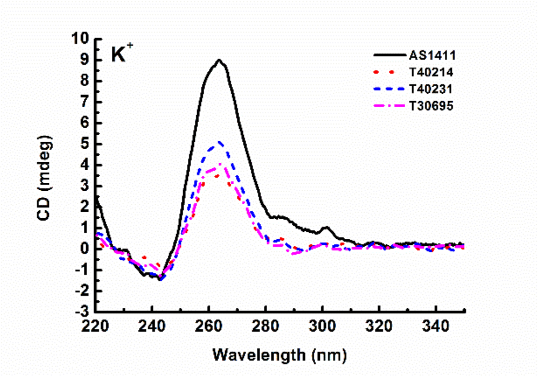
K+
AS1411
T40214
T40231
T30695
CD (mdeg)
Wavelength (nm)

e

Table S1. Nucleotide sequences used for the CD spectral study

No	name	sequence	length	topology in K^+	topology in Na^+	ref.
1	C-MYC	TGGGGAGGGTGGGGAGGGTGGGGAAGG	27	parallel	-	[35, 36]
2	c-kit2	CGGGCGGGCGCGAGGGAGGGG	21	parallel	-	[38]
3	RET20T	GGGGCGGGGCGGGGCGGGGT	20	parallel	-	[39]
4	RET21mer	GGGGCGGGGCGGGGCGGGGGT	21	parallel	-	[39]
5	93DEL	GGGGTGGGAGGAGGGT	16	parallel	-	[40]
6	Normal Form1	TAGGGTTAGGGTTAGGGTTAGGG	30	hybrid	basket anti	[41-43]
7	Normal Form2	TAGGGTTAGGGTTAGGGTTAGGGTT	33	hybrid	basket anti	[41-43]
8	Two Forms	TAGGGTTAGGGTTAGGGTTAGGGT	24	hybrid	basket anti	[41-43]
9	A-Tel21	AGGGTTAGGGTTAGGGTTAGGG	22	hybrid	basket anti	[44]
10	N-22	GGGATGGGACACAGGGGACGGG	22	hybrid	-	[45]
11	PFV34	CAGGGTTAAGGGTATAACTTTAGGGGTTAGGGTT	34	hybrid	-	[47]
12	Oxy3.5	GGGGTTTTGGGGTTTTGGGGTTTTGGGG	28	-	basket anti	[48]
13	GRO20A	GGTTTTGGTTTTGGTTTTGG	20	-	basket anti	[32]
14	TBA	GGTTGGTGTGGTTGG	15	chair anti	-	[49, 50]
15	Bom19	TAGGTTAGGTTAGGTTAGG	19	chair anti	-	[33]
16	T30695	GGGTGGGTGGGTGGGT	16	chair anti	-	[34, 51]
	T30695	GGGTGGGTGGGTGGGT	16	Parallel	-	[29]
17	T40214	GGGCGGGCGGGCGGGC	16	chair anti	-	[52]
	T40214	GGGCGGGCGGGCGGGC	16	Parallel	-	[29]
18	GRO23A	GGGGTTGGGGTGTGGGGTTGGGG	23	-	mixed basket/chair anti	[32]

Table S1. The sequences studied in this manuscript

No	name	sequence	length	topology in K^+	topology in Na^+	ref.	No
1	C-MYC	TGGGGAGGGTGGGGAGGGTGGGGAAGG	27	parallel	-	[35, 36]	1
2	c-kit2	CGGGCGGGCGCGAGGGAGGGG	21	parallel	-	[38]	2
3	RET20T	GGGGCGGGGCGGGGCGGGGT	20	parallel	-	[39]	3
4	RET21mer	GGGGCGGGGCGGGGCGGGGGT	21	parallel	-	[39]	4
5	93DEL	GGGGTGGGAGGAGGGT	16	parallel	-	[40]	5
6	Normal Form1	TAGGGTTAGGGTTAGGGTTAGGG	30	hybrid	basket anti	[41-43]	6
7	Normal Form2	TAGGGTTAGGGTTAGGGTTAGGGTT	33	hybrid	basket anti	[41-43]	7
8	Two Forms	TAGGGTTAGGGTTAGGGTTAGGGT	24	hybrid	basket anti	[41-43]	8
9	A-Tel21	AGGGTTAGGGTTAGGGTTAGGG	22	hybrid	basket anti	[44]	9
10	N-22	GGGATGGGACACAGGGGACGGG	22	hybrid	-	[45]	10
11	PFV34	CAGGGTTAAGGGTATAACTTTAGGGGTTAGGGTT	34	hybrid	-	[47]	11
12	Oxy3.5	GGGGTTTTGGGGTTTTGGGGTTTTGGGG	28	-	basket anti	[48]	12
13	GRO20A	GGTTTTGGTTTTGGTTTTGG	20	-	basket anti	[32]	13
14	TBA	GGTTGGTGTGGTTGG	15	chair anti	-	[49, 50]	14
15	Bom19	TAGGTTAGGTTAGGTTAGG	19	chair anti	-	[33]	15
16	T30695	GGGTGGGTGGGTGGGT	16	chair anti	-	[34, 51]	16
	T30695	GGGTGGGTGGGTGGGT	16	Parallel	-	[29]	
17	T40214	GGGCGGGCGGGCGGGC	16	chair anti	-	[52]	17
	T40214	GGGCGGGCGGGCGGGC	16	Parallel	-	[29]	
18	GRO23A	GGGGTTGGGGTGTGGGGTTGGGG	23	-	mixed basket/chair anti	[32]	18
